# Rare Genetic Variants in Complement Factor I Lead to Low FI Plasma Levels Resulting in Increased Risk of Age-Related Macular Degeneration

**DOI:** 10.1167/iovs.61.6.18

**Published:** 2020-06-09

**Authors:** Thomas M. Hallam, Kevin J. Marchbank, Claire L. Harris, Clive Osmond, Victoria G. Shuttleworth, Helen Griffiths, Angela J. Cree, David Kavanagh, Andrew J. Lotery

**Affiliations:** 1 Complement Therapeutics Research Group, Translational and Clinical Research Institute, Newcastle University, Newcastle upon Tyne, United Kingdom; 2 National Renal Complement Therapeutics Centre, Royal Victoria Infirmary, Newcastle upon Tyne, United Kingdom; 3 Clinical and Experimental Sciences, Faculty of Medicine, University of Southampton, Southampton, United Kingdom; 4 MRC Lifecourse Epidemiology Unit, University of Southampton, Southampton, United Kingdom

**Keywords:** age-related macular degeneration, complement factor I, aqueous humor, complement system, rare genetic variants

## Abstract

**Purpose:**

Rare genetic variants in complement factor I (*CFI*) that cause low systemic levels of the protein (FI) have been reported as a strong risk factor for advanced age-related macular degeneration (AMD). This study set out to replicate these findings.

**Methods:**

FI levels were measured by sandwich ELISA in an independent cohort of 276 patients with AMD and 205 elderly controls. Single-nucleotide polymorphism genotyping and Sanger sequencing were used to assess genetic variability.

**Results:**

The median FI level was significantly lower in those individuals with AMD and a rare *CFI* variant (28.3 µg/mL) compared to those with AMD without a rare *CFI* variant (38.8 µg/mL, *P* = 0.004) or the control population with (41.7 µg/mL, *P* = 0.0085) or without (41.5 µg/mL, *P* < 0.0001) a rare *CFI* variant. Thirty-six percent of patients with AMD with a rare *CFI* variant had levels below the fifth percentile, compared to 6% in controls with *CFI* variants. Multiple regression analyses revealed a decreased FI level associated with a rare *CFI* variant was a risk factor for AMD (early or late AMD: odds ratio [OR] 12.05, *P* = 0.03; early AMD: OR 30.3, *P* = 0.02; late AMD: OR 10.64, *P* < 0.01). Additionally, measurement of FI in aqueous humor revealed a large FI concentration gradient between systemic circulation and the eye (∼286-fold).

**Conclusions:**

Rare genetic variants in *CFI* causing low systemic FI levels are strongly associated with AMD. The impermeability of the Bruch's membrane to FI will have implications for therapeutic replacement of FI in individuals with *CFI* variants and low FI levels at risk of AMD.

Age-related macular degeneration (AMD) is the leading cause of irreversible vision loss in the developed world, and current estimates predict that around 196 million people will be afflicted with the disorder by 2020, rising to 288 million by 2040.^1^ AMD is characterized by lipoproteinous drusen deposits that form in the subretinal space and the recruitment of immune cells, such as microglia and macrophages,^2^^,^^3^ during the early and later stages of the disorder. Degeneration and geographic atrophy (GA) of the retinal pigment epithelium (RPE) and photoreceptors occur in the macula of the retina in advanced dry AMD, while advanced wet AMD involves angiogenesis in the choroid and subsequent choroidal neovascularization. As of yet, there is no effective treatment for dry AMD, and while anti-VEGF therapy has been reasonably successful in the short term, vision gains are not maintained at 5 years after starting treatment.^4^ Ultimately, many patients progress to GA, for which there is currently no treatment. Further, anti-VEGF treatments require up to monthly intravitreal injections, which is difficult to maintain long term. Personalized therapeutics for AMD that might delay progression to the late stages of the disease would be highly beneficial. However, for safety and economic reasons, such treatments will need to be stratified to those patients most likely to benefit from them.

The complement system is an ancient innate immune response system with three main pathways: the classical (CP), mannose-binding lectin, and alternative pathways (AP). Studies over the past decade or so have implicated the complement system, and more specifically the AP,^5^ in being involved in AMD pathologic progression. Patients with genetic variations in the genes that encode the endogenous inhibitors of the alternative pathway, FH and FI, are considered at greater risk of developing AMD.^6^^–^^13^

FI, which is encoded by the *CFI* gene on ch4q25, is a serine protease and an endogenous inhibitor of the complement pathway.^14^^,^^15^ It functions by cleaving the α’ chains of C3b and C4b in the presence of cofactors: FH for C3b,^16^^,^^17^ C4 binding protein (C4BP) for C4b,^18^^,^^19^ and CD46 and complement receptor 1 (CR1; CD35) for both.^20^^,^^21^ This limited proteolytic cleavage of C3b and C4b regulates both the AP and the CP; however, as the AP is a positive feedback loop initiated by C3b deposition on a target, it is C3b's inactivation that is the critical regulatory function of FI.

Complete deficiency of FI in humans results in unchecked turnover of the AP, a consumptive C3 deficiency, and predisposition to infection with encapsulated organisms (reviewed in Lachmann^22^). These individuals are not, however, at risk of immunopathology, due to the lack of generation of iC3b, in contrast to individuals with FI haploinsufficiency, which has been associated with atypical hemolytic uremic syndrome (aHUS), albeit with low penetrance.^23^

Association studies first identified common single-nucleotide polymorphisms (SNPs) associated with AMD risk, suggesting a role for *CFI* in disease pathogenesis (e.g. rs11726949, odds ratio [OR] ∼1.6).^9^ Subsequently, next-generation sequencing studies demonstrated an increased burden of rare *CFI* variants in AMD,^12^^,^^13^ with a much increased odds ratio (OR ∼3.6), earlier age of onset, and altered disease progression.^24^ To assess the functional consequences of these variants, Kavanagh et al.^25^ assessed the FI antigenic levels in serum in a single AMD cohort and demonstrated that rare variants in *CFI* causing low levels (type I variants) are the predominant driver of AMD risk (OR 13.6).

In addition to genetic studies linking impaired complement regulation by FI to AMD, amyloid β, a constituent of drusen deposits in AMD, has been suggested to interact with FI, impairing its function in both AMD and Alzheimer disease.^26^^,^^27^

These genetic and in vitro studies suggest a role for impaired regulation of the complement system through FI in AMD. In this study, we replicate, in an independent cohort, the association of rare *CFI* variants causing low plasma FI levels as a strong risk factor for AMD (OR 12).

## Methods

### Patient Sample Collection and Selection

The Southampton AMD cohort contains 577 patients with AMD and 651 elderly controls. Cases and controls were examined and diagnosed by AJL as previously described,^11^ before the cases were classified into the appropriate subcategories as per the age -related eye disease studies (AREDS) system.^28^ Consent was obtained in accordance with the Declaration of Helsinki and was approved by South West Hampshire Local Research Ethics Committee (374/02/t and 150/03/t). Informed consent was obtained from all subjects, and all methods were carried out in accordance with the relevant guidelines and regulations of Research Ethics Committees. Late (wet, dry, or mixed) and early disease patients were later separated for part of the statistical analysis. All patients were analyzed by SNP-chip genotyping, and FI levels were measured in 276 AMD cases and 205 elderly controls, selected based on availability of plasma and additional patient information such as C-reactive protein (CRP) level, smoking status, and body mass index.

Aqueous fluid was extracted, along with corresponding serum, from patients in a distinct cohort of 16 cases and 14 controls by AJL during cataract surgery and stored at –80°C before FI levels were measured.

### Measurement of Factor I Levels

Ninety-six well-plates (Maxisorp; ThermoFisher, Waltham, MA, USA) were coated with 2 µg/mL capture antibody (sheep anti-FI polyclonal Ab, Abcam 8843; Abcam, Cambridge, UK) in a sodium bicarbonate buffer and incubated overnight at 4°C. The plates were then washed three times with PBST (0.1% Tween in phosphate-buffered saline) before 50 µL of blocking buffer solution (1% BSA in PBST) was added to each well. Again, the plates were washed before 50 µL of sample and standard was added to wells in triplicate and duplicate, respectively. The standard was made up of 8 1:2 serial dilutions from 1 µg/mL purified FI (A138; CompTech, Tyler, TX, USA). The plates were incubated for 1 hour at room temperature before further stages of washing, addition of 50 µL detection antibody (Ab) (mouse monoclonal anti-FI Ab, OX-21, produced in house at an initial concentration of 1 mg/mL, diluted 1/1000 in blocking buffer), washing with PBST, and addition of 50 µL tertiary Horseradish Peroxidase (HRP)-conjugated donkey anti-mouse secondary Ab (715-035-150JIR; Stratech, Ely, UK) (1/2000 in blocking buffer). A final wash step was performed before 100µL tetramethylbenzidine solution was added to each well. After incubation for 5 minutes, 100µL of stop solution (1 M H_2_SO_4_) was added and the plates were immediately read at 450nm absorbance using a Floustar Optima plate reader (BMG Labtech). FI levels of patients were measured using the ELISA described, and results were read on the plate reader, which had integrated interpolation software for immediate results given from the computer-generated standard curve. Calculated intraplate and interplate percent coefficient of variation were 3.18 and 14.87, respectively, for a normal control patient across all 25 plates in triplicate. The fifth percentile plasma FI cutoff of 23.69 µg/mL was calculated using the levels of the 205 control patients that were measured. For measurements of aqueous fluid, the standard curve was extended from 8 to 10 1:2 dilutions, with an average lower limit of detection of 0.54 ng/mL and lower limit of quantification (LLOQ) of 0.87 ng/µL, as calculated by interpolation of the blank + SD of two blank replicates per plate multiplied by 3 and 10, respectively. Serum samples were diluted 1:1000, while aqueous fluid was diluted 1:80 in order to fit the detectable range; the concentration of the lowest test sample was never below the LLOQ for an individual plate.

### Genetic Analysis

SNP-chip genotyping of the Southampton AMD case-control cohort was performed as part of the sequencing of the AMD-European Union-Johns Hopkins University (EU-JHU) cohort by the procedures outlined in the study by the AMD Gene Consortium.^29^ In those individuals with plasma FI levels below the fifth percentile, Sanger sequencing^30^ and multiplex ligand-dependent probe amplification^31^ were also performed as described. Rare genetic variants were defined as Minor Allele Frequency (MAF) <1% in the cohort.

### Statistics

**Figure 1.**
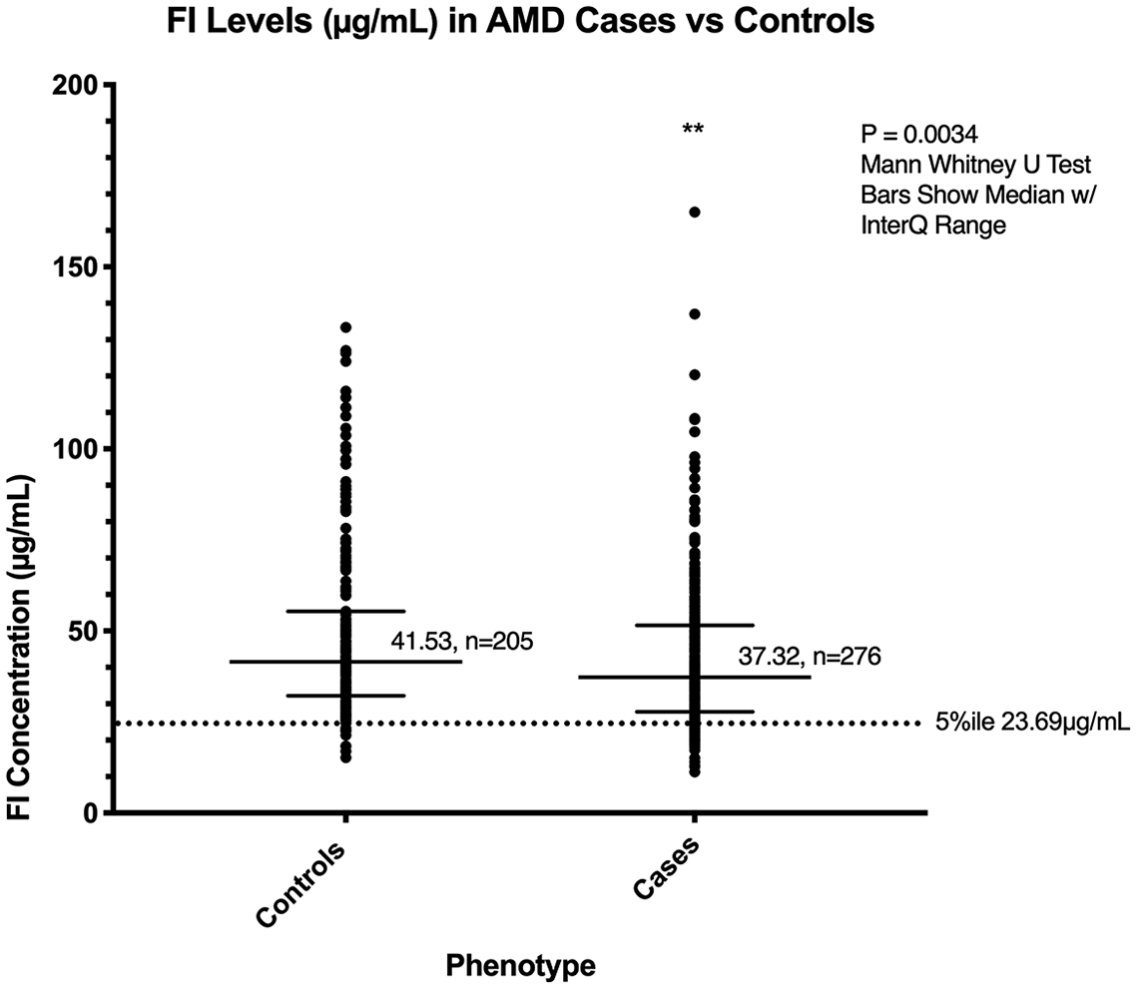
Median factor I levels in patients with AMD and controls. A sandwich ELISA was used to measure the FI level (µg/mL) in the plasma of patients in the Southampton AMD cohort. These levels were measured in triplicate and calculated as an interpolation of OD values on a computer-generated standard curve resulting from 1:2 dilutions of purified FI starting from 1 µg/mL and ending at 0.004 µg/mL. The median level was significantly lower in cases compared to controls (*P* = 0.0034, interquartile range shown, for 276 cases and 205 controls). A low fifth percentile of 23.69 µg/mL was calculated from the healthy control cohort only and is indicated by the *dotted line*. **P < 0.01.

**Figure 2.**
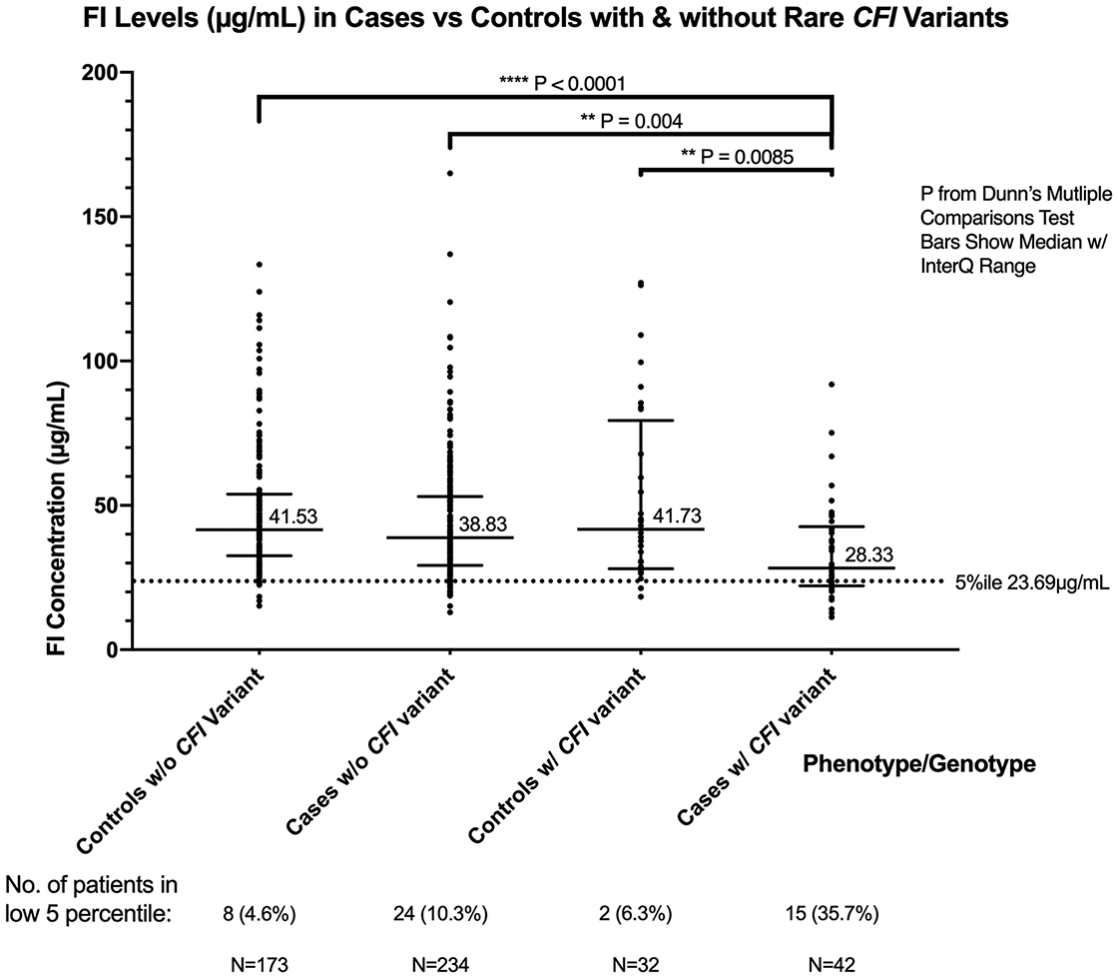
Plasma factor I levels in patients with AMD and controls by rare *CFI* variant status. FI levels (in µg/mL) in plasma were significantly lower in AMD cases with *CFI* variants when compared to all other groups. *P* values were determined using Dunn's multiple comparisons test. The median and interquartile range are shown by *bars*. The fifth percentile cutoff point is indicated by the *dotted line*. *P < 0.05. **P < 0.01. ****P < 0.0001.

All standard statistical tests, apart from those in Figure 4, were performed using GraphPad Prism version 7.0a for Mac (GraphPad Software, La Jolla, CA, USA). The differences between FI levels in patients were compared using a Mann-Whitney *U* test (Fig. 1) and Dunn's multiple comparisons test (Fig. 2), and the common SNP analysis was performed using the Mann-Whitney *U* test comparing homozygous noncarriers to heterozygous patients (see Supplementary Figs. S2 and S3). The single-variant association study was performed using the GraphPad Prism 2 × 2 contingency table and Fisher's exact test. For Figure 4, multiple logistic regression models were run to analyze the patient data using IBM SPSS version 25. A basic χ^2^ test was used to interrogate differences between each model. Standard linear regression was used to calculate associations between age, sex, smoking status, CRP level, FI level, and AMD in the first instance. However, only age and CRP level were used to give adjusted ORs in the final models. CRP and FI plasma level values were converted to the log (ln) scale in order to normalize the distributions. Significance was defined as *P < 0.05, **P < 0.01, ***P < 0.001, and ****P < 0.0001.

## Results

### Plasma Factor I Levels

The FI plasma level was measured in 276 patients with AMD and 205 elderly controls. The median FI level was lower in cases compared to controls (37.2 vs. 41.53 µg/mL, *P* = 0.0034). A low plasma FI cutoff point was established by taking the lower fifth percentile cutoff points in the control cohort (23.69 µg/mL), as shown in Figure 1.

### Genetic Analysis

Genetic analysis of the cohort revealed 23 distinct rare *CFI* variants in 42 cases and 32 controls. SNP-chip sequencing elucidated 20 rare *CFI* variants, including G119R, G261D, R406H, K441R, and P553S changes, in a total of 79 patients for some of whom there was no available plasma. Sanger sequencing of the *CFI* gene, which was performed on the DNA of patients with FI levels below the fifth percentile cutoff and no previously SNP-chip-elucidated *CFI* variant, enabled us to identify three new variants in four patients with AMD with low levels (N245S, V412M, and G362A; G362A found in two patients).

The relationship between FI level and two common SNPs was analyzed in this cohort, rs11726949 and rs9998151 (Supplementary Figs. S1 and S2). There was a small but significant decrease in FI level when comparing the median of carriers of rs11726949 in heterozygosity compared to noncarriers (Supplementary Fig. S1), although the relative rarity of rs11726949 in our cohort requires that this result is validated in additional cohorts.

### Analysis of FI Levels in Rare *CFI* Variant Carriers

**Figure 3.**
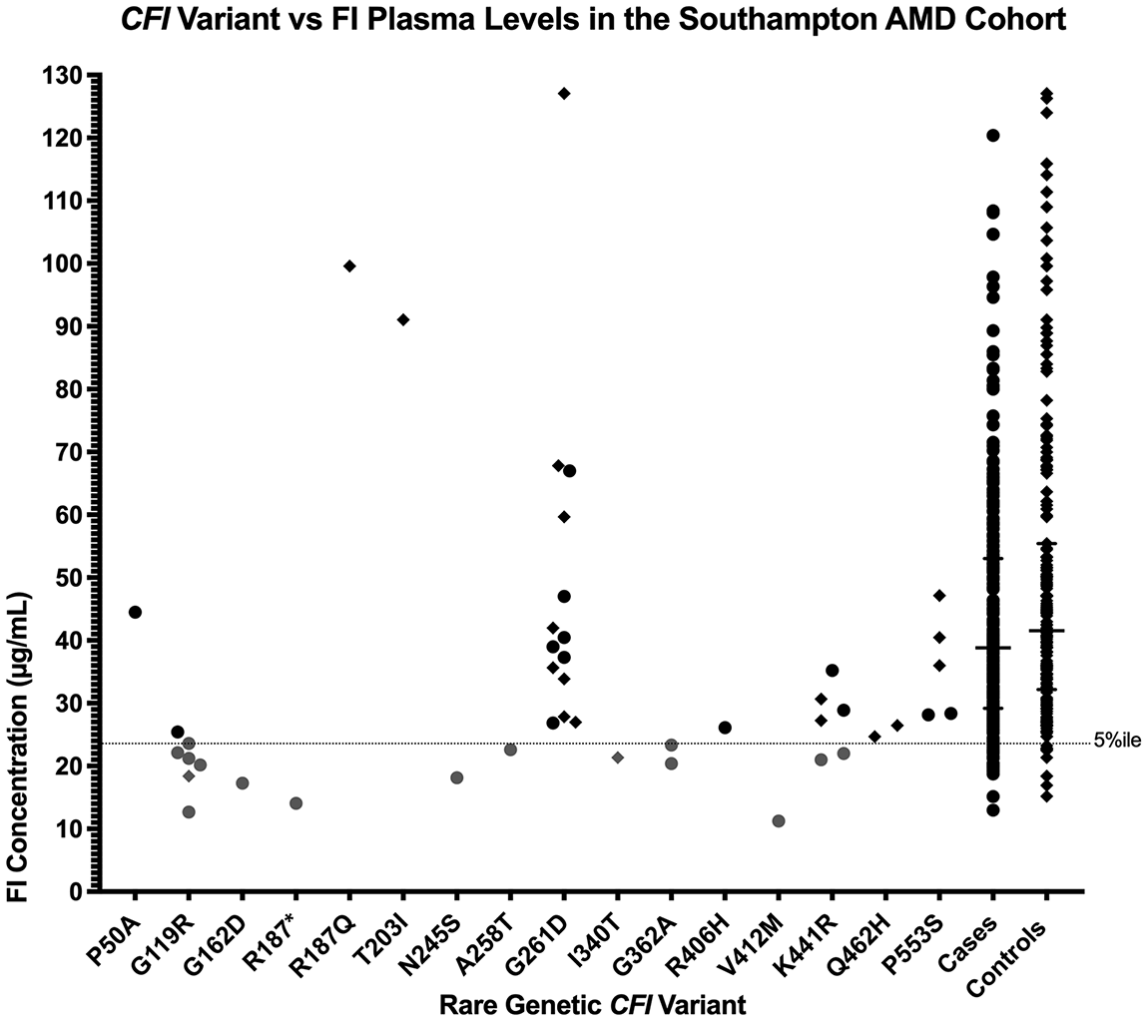
*CFI* rare genetic variant versus factor I plasma level. The nonsynonymous genetic variants in *CFI* identified in the cohort are plotted along the x-axis, while the corresponding plasma FI level (µg/mL) in each patient (case (●), or control (◆)) carrying the given variant in the cohort is displayed on the y-axis. The low fifth percentile is indicated by the *dotted line*.

To probe the finding of lower FI levels in the AMD population, analysis was undertaken according to *CFI* genotype. The median FI level was significantly lower in those individuals with AMD and a rare *CFI* variant (28.3 µg/mL) compared to those with AMD without a rare *CFI* variant (38.8 µg/mL, *P* = 0.004) or the control population with (41.7 µg/mL, *P* = 0.004) or without (41.5 µg/mL, *P* < 0.0001) a rare *CFI* variant (Fig. 2). Seventeen patients out of 74 with a rare variant had levels below the fifth percentile, and 36% of patients with AMD with a rare *CFI* variant had levels in the fifth percentile, compared to 6% in controls with *CFI* variants. Ten percent and 5% of cases and controls, respectively, had low levels without a rare *CFI* variant. Sixteen different rare, nonsynonymous *CFI* genetic variants were identified. Of these variants, FI levels below the fifth percentile were seen in patients carrying G119R, G162D, R187*, N245S, A258T, I340T, G362A, V412M, and K441R. One patient out of seven with G119R and four patients out of six with K441R had levels above the fifth percentile (Fig. 3^4^).

**Figure 4.**
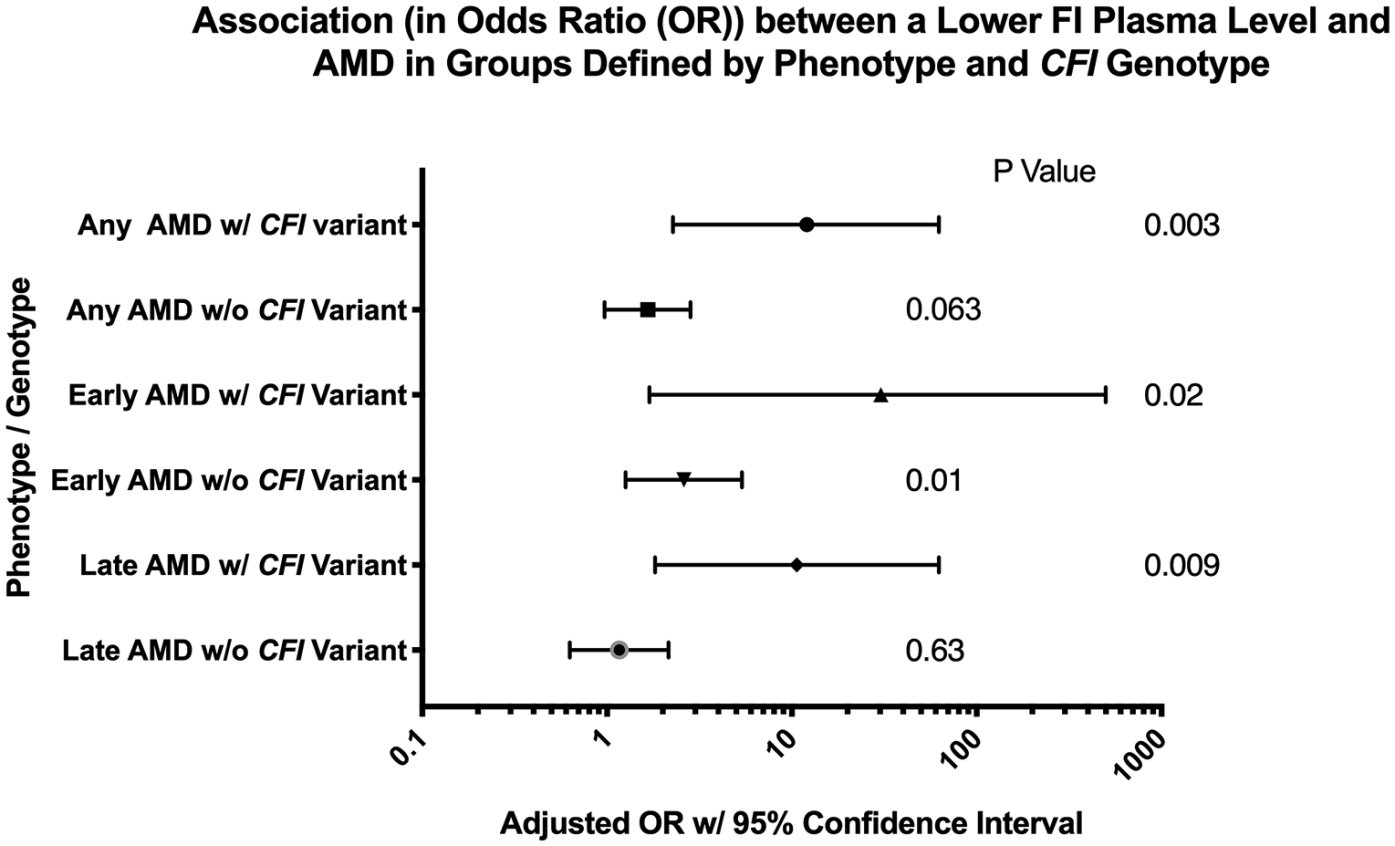
The association between factor I level, rare *CFI* variant status, and AMD. Plotted are odds ratios and 95% confidence intervals from six logistic regression models used to predict AMD (any, early, or late) according to whether the patient had a rare *CFI* variant. Each model is adjusted for age and CRP level, and the odds ratio is per one natural log (ln) unit decrease in FI level (µg/mL). The odds ratios are similar for any, early, and late AMD within the same genotype groups (i.e., with *CFI* variant or without *CFI* variant). However, the odds ratios for any and late AMD in the groups with a rare *CFI* variant (OR 12.05, *P* = 0.003 and OR 10.6, *P* = 0.009, respectively) are significantly higher than those in the groups without a *CFI* variant (OR 1.66, *P* = 0.06 and OR 1.16, *P* = 0.63, respectively). The test for the difference in these values, performed using a 1-*df* chi-squared test has χ^2^ = 4.93, *P* = 0.03 and χ^2^ = 5.378, *P* = 0.02 for the any AMD cohort and the late AMD cohort, respectively. Statistical significance was reached at *P < 0.05.

### Single Rare *CFI* Variant Association With AMD

In order to identify direct associations between variants in our cohort and AMD, single-variant disease association analysis was performed (Table) on rare nonsynonymous variants that occurred five or more times in the SNP-chip data for our cohort. Only the G119R variant associated with low FI levels was identified as being significantly associated with AMD in the Southampton AMD cohort (seven cases and one control, *P* = 0.026). K441R was identified in four cases and two controls but did not reach a level of significant association with AMD in the cohort. K441R was associated with low levels (two cases, 33%), and both of these low FI level K441R carriers had early AMD. The variants P553S and G261D were found in more controls than cases, although not significantly so (Table). The previously described protective R406H variant was seen only twice in the entire cohort, in one case and one control.

**Table.** Single-Variant Association of Rare *CFI* Variants in the Southampton AMD Cohort

Rare *CFI* Variant	Cases, *n*	Controls, *n*	*P* Value
G119R	7	1	0.0296
K441R	4	2	0.4283
P553S	2	3	1
G261D	6	15	0.1214

Single-variant association studies were performed using GraphPad prism v7, with a 2 × 2 contingency table, Fisher's exact test, and a two-tailed *P* value. The association between AMD incidence and the four rare *CFI* variants recurring five or more times in the Southampton cohort, as per the SNP-chip sequencing only, is displayed here. Only the G119R variant reached statistical significance (*P* = 0.0296), which was defined as *P < 0.05.

### Regression Analyses

Finally, multiple regression analyses were performed using available patient data, and statistical models were created to calculate ORs for any, early, and late AMD manifestation in patients per decrease in FI level by one unit on the log (ln) scale (any: OR 1.66, *P* = 0.063; early: OR 2.60, *P* = 0.01; late: OR 1.16 *P* = 0.63), which were greatly amplified if the patient had a rare *CFI* variant (any: OR 12.05, *P* = 0.03; early: OR 30.30, *P* = 0.02; late: OR 10.64, *P* = 0.009). In fact, only risk of early AMD was significantly affected by low FI levels alone. To consolidate this, it was further calculated that in any AMD and late AMD groups, the ORs produced by the models were in fact statistically significantly increased by having a rare genetic *CFI* variant (χ^2^ = 4.93, *P* = 0.03 and χ^2^ = 5.378, *P* = 0.02 for the any AMD cohort and the late AMD cohort, respectively). This clearly illustrates the deleterious nature of type I *CFI* variants, with patients far more likely to develop disease with a lower FI level and a *CFI* variant*.*

In addition to the FI level analyses, it was also elucidated that high CRP levels were associated with late, but not early, AMD risk (OR 1.3, *P* = 0.03) in patients with no rare *CFI* variant and that being male was linked with a patient having a lower FI level (*P* = 0.047). We also found that having a rare, nonsynonymous *CFI* variant was greatly associated with having a lower FI level in all individuals (*P* < 0.0001).

### Aqueous Factor I Levels

**Figure 5.**
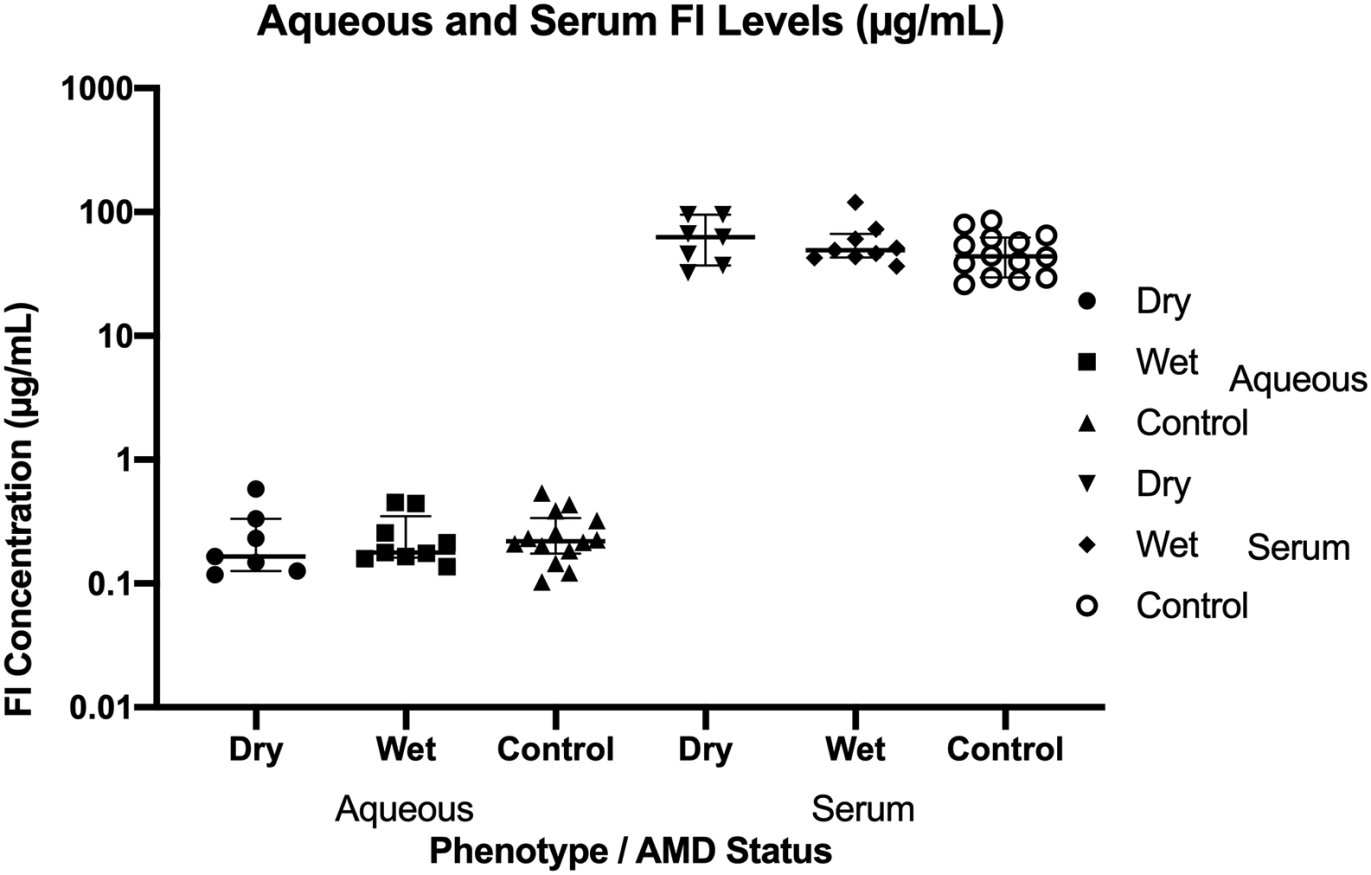
Concurrent serum and aqueous factor I levels measurement. FI concentrations (µg/mL) were measured in matched aqueous fluid and serum samples in 16 cases (without rare genetic variants) and 14 controls. There were no differences between the mean or median (median displayed with interquartile range, as shown by *bars*) FI levels in each group in this instance, but the mean values demonstrated a concentration gradient between serum and aqueous humor. The mean concentration gradient [FI Conc. in Serum/FI Conc. in Aqueous] is 322.5-fold (SEM ± 52.07) in cases and 243.6-fold (SEM ± 41.83) in controls. This implies that the blood retinal barrier acts as an impermeable blockade to FI and suggests that the majority of FI present in the eye is secreted by the RPE (or other components of the retina) itself.

We concurrently measured serum and aqueous FI levels in 16 AMD cases (7 dry and 9 wet AMD) and 14 control patients. In this small cohort, there was little difference in the mean FI serum levels in dry (62.08 µg/mL), wet (57.98 µg/mL), or control (48.58 µg/mL) patients. Likewise, there were no differences in mean aqueous FI levels between dry (0.24 µg/mL), wet (0.24 µg/mL), or control (0.26 µg/mL) patients (Fig. 5). However, there was a large concentration gradient between the serum and aqueous in both cases (mean = 322.5-fold) and controls (mean = 243.6-fold), ranging from 55.2- to 803.5-fold (Supplementary Fig. S1).

## Conclusions

This study validates, in an independent cohort, the findings of Kavanagh et al.,^25^ demonstrating that rare genetic variants in *CFI* resulting in low FI plasma levels are a strong risk factor for AMD. The median FI plasma level in patients with AMD was significantly lower than in the control population, but this was largely driven by rare genetic variants in the *CFI* gene, with a significantly lower median level identified in those patients with AMD with variants compared to those without. Further, 36% of patients with AMD with a rare *CFI* variant had low levels compared to 6% of the control population with a rare *CFI* variant, remarkably similar figures to those described in Kavanagh et al.^25^ (42% vs. 6%).

Multiple regression analyses demonstrated a significant association between early AMD (OR 2.6, *P* = 0.01) and a decrease in FI level (per one unit on the ln scale) in patients with no *CFI* variant, whereas there was a trend toward significance in the any AMD group (OR 1.66, *P* = 0.063). Statistical models that combined a decrease in FI level and a rare *CFI* variant all showed significant AMD ORs—any AMD (OR 12), early AMD (OR 30.3), and late AMD (OR 10.6)—and these ORs for any and late AMD were significantly higher than those in the respective models without *CFI* variants. These data suggest that a low FI level associated with a rare *CFI* variant is a strong risk factor for AMD. This is in keeping with the findings of Kavanagh et al.,^25^ who demonstrated an OR of 13.6 for advanced AMD with a rare *CFI* variant and low FI level, which would best compare with the late AMD OR of 10.6 in this study, albeit that a different modeling was used for calculation of the affect.

Regression analysis also demonstrated that a higher CRP level is significantly associated with late AMD in patients without rare *CFI* variants (OR 1.3, *P* = 0.03). Previous studies have shown an association between high CRP levels and AMD.^32^ CRP level changes do not reach significance in OR in any other subgroup model in this study (early, late, or any AMD with a *CFI* variant or early or any AMD without a *CFI* variant).

Nine separate variants were seen in association with low FI levels: G119R, G162D, R187*, N245S, A258T, I340T, G362A, V412M, and K441R. Five of these variants have previously been associated with low levels of FI in either aHUS (G119R, G162D, K441R)^33^^–^^35^ or AMD (G119R, G162D, R187*, A258T, K441R)^25^ and thus can be categorized as type I variants.

In our study, the patient with P50A had normal levels of FI, and although it has also been identified in patients with aHUS with normal FI levels,^36^ this variant was reported associated with low levels in Kavanagh et al.^25^ In contrast, patients with V412M, G362A, and I340T have been reported to have normal levels but are low in our study.^25^^,^^34^^,^^37^ Of note, previous functional analysis of I340T demonstrated absent activity.^37^ FI is an acute phase protein with cytokines known to increase secretion, and plasma levels vary widely in the normal population. As a limitation to the study, rare variants known to cause impaired secretion can be masked by increased secretion of protein made from the normal allele. This can be clearly seen in the case of G119R, the commonest type I mutation in AMD. Despite being consistently associated with low FI levels, in our study, one individual out of seven had levels just above the fifth percentile. Likewise, patients with K441R may have low or normal levels as reported in Kavanagh et al.^25^ Thus, although a low FI level is highly associated with rare genetic *CFI* variants and AMD, physiologic variation can mask a type I mutation. N245S is to date an entirely novel rare *CFI* variant, which was detected by Sanger sequencing and observed associated with a very low FI level in a patient with AMD in the cohort. V412M, which has been reported recently in familial AMD with severe phenotype,^38^ was found associated with a very low FI level, also in a single patient with AMD in our cohort.

In our study, we also noted that in patients with AMD without rare *CFI* variants, 10.3% had low FI levels compared to 4.6% in the control population. Although it is possible that other genetic factors determining FI expression were not examined in our study, it maybe that sequestration of FI at sites of complement activation may account for these low levels. It has been noted in Systemic Lupus Erythematosus (SLE) that factor I serum levels are lower during SLE active phases compared to recessive phases in patients, suggesting possible complement-mediated FI consumption during disease.^39^

In a small subgroup of patients with AMD and controls without rare genetic mutations in *CFI*, we were able to sample serum and aqueous humor simultaneously. FI levels identified in the aqueous humor were similar to those in previously published work by Schick et al.^40^ Although the numbers were too small to see significant differences between dry AMD, wet AMD, and control populations in either serum or aqueous humor, there was a striking concentration gradient between serum and aqueous across all groups (average concentration gradient ∼286-fold). Such a gradient suggests compartments separated by an FI-impermeable barrier. This is in keeping with Clark et al.,^41^ who demonstrated that FI cannot pass through the Bruch's membrane/RPE layer of the outer blood retinal barrier (BRB), which under normal conditions acts to maintain immune privilege in the eye, constraining proteins such as factor I and full-length factor H within the choroid and subretina, while supporting the delicate processes of the photoreceptors and neural retina. Such a finding is of key importance if pharmacologic supplementation of FI is to be trialed for AMD as intravenous therapy would fail to gain entry to the eye. The importance of local complement synthesis in the eye in AMD has been suggested previously by ourselves and others. We demonstrated in liver transplant patients that the recipient *CFH* risk genotype was associated with AMD but not donor *CFH* genotype, suggesting the role of intraocular complement activation.^42^ Further, experiments by Anderson et al.^43^ have identified FI expression and localization at cells of the neuroretina and RPE at higher levels relative to the choroid, whereas factor H appears to be expressed more highly at the choroid. Further, Li et al.^44^ illustrated that *CFI* is more highly expressed in the macular RPE compared to the neural retina or cells of the peripheral retina, whereas measurements of other alternative pathway proteins showed no region-specific distribution differences. Together, these data suggest an important role for complement inhibition within the inner and outer macular, lending particular credence to a role in AMD, and show that cells of the eye have the ability to express and secrete the majority of complement components and regulators, including *CFI*, to varying degrees. We therefore hypothesize that being heterozygous for a type I *CFI* variant would lead to haploinsufficiency in the microenvironment of the eye, resulting in higher levels of alternative pathway activity. These data suggest that patients with type I *CFI* variants may benefit from complement inhibitory therapy, but the impermeability of the eye to FI suggests that intraocular administration may be required.

In summary, we demonstrate that rare genetic *CFI* variants causing low FI levels are a substantial risk factor for AMD and identify several individual rare, type I *CFI* variants in patients with AMD with low FI levels. This replicates the findings of Kavanagh et al.^25^ in an independent cohort and lends further support to the hypothesis that FI supplementation would be efficacious in the prevention of AMD progression. The mode of delivery will need to take into account the concentration gradient for FI across the BRB due to its impermeability to FI.

## Supplementary Material

Supplement 1
