# Supplementary material for: Rare Genetic Variants in Complement Factor I Lead to Low FI Plasma Levels Resulting in Increased Risk of Age-Related Macular Degeneration
Source: Invest Ophthalmol Vis Sci. 2020 Jun 9;61(6):18. doi: 10.1167/iovs.61.6.18 (PMC7415286; doi:10.1167/iovs.61.6.18)
Supplement: Supplement 1 [file iovs-61-6-18_s001.docx]

**
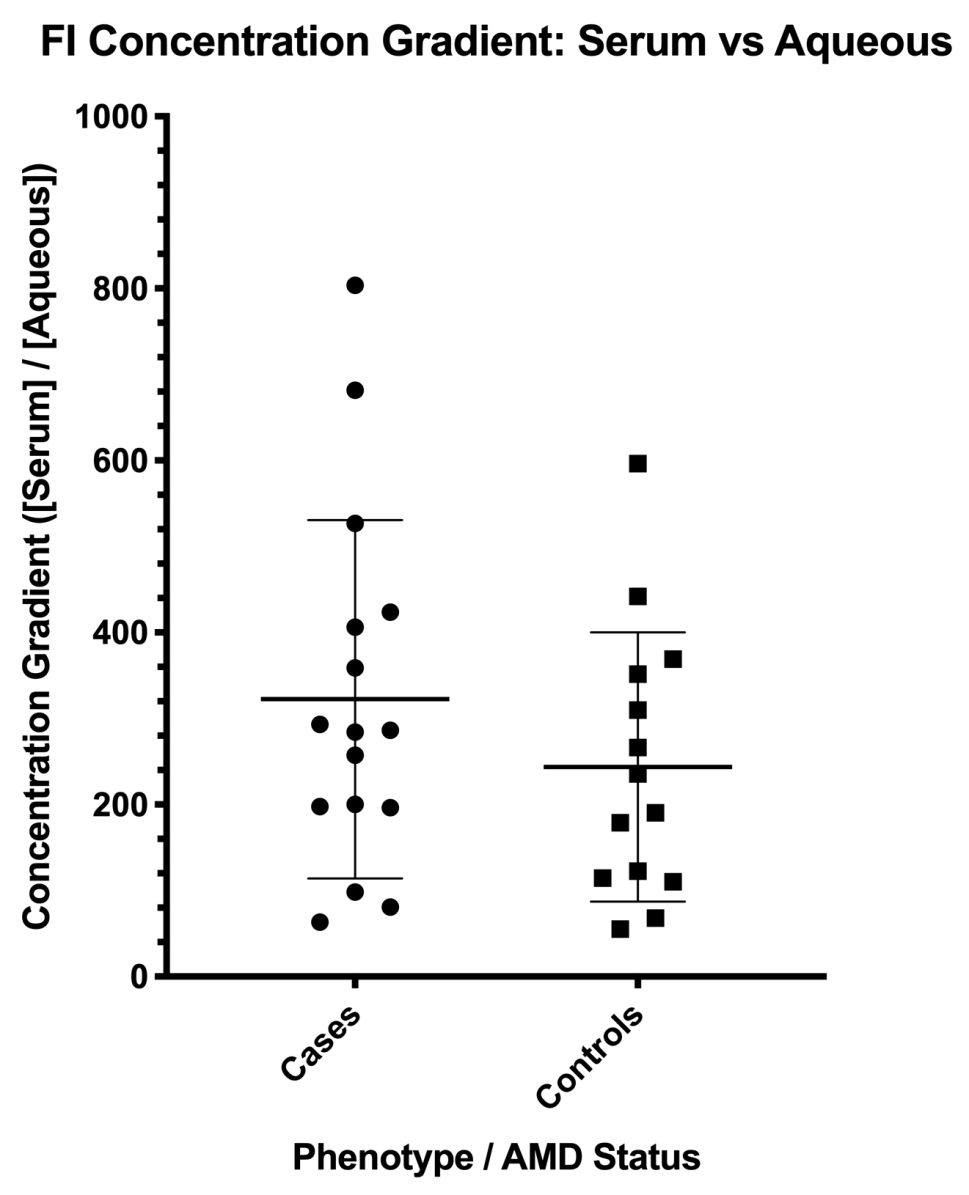
**

**Supplementary figure 1** - Plotted are the FI concentration gradients between the matched aqueous and serum samples of both AMD cases and control patients in the small aqueous cohort. Mean with SD is indicated by bars. Cases (322.5-fold) had a slightly larger mean gradient than controls (243.6-fold). This data shows that there is a huge range in the concentration gradient from 55.2-fold to 803.5-fold. This reinforces the idea of a highly selective barrier to FI at the blood-retinal barrier and as such local FI secreted by cellular components of the retina will be critical in combatting downstream effects of complement activation in the eye.

**
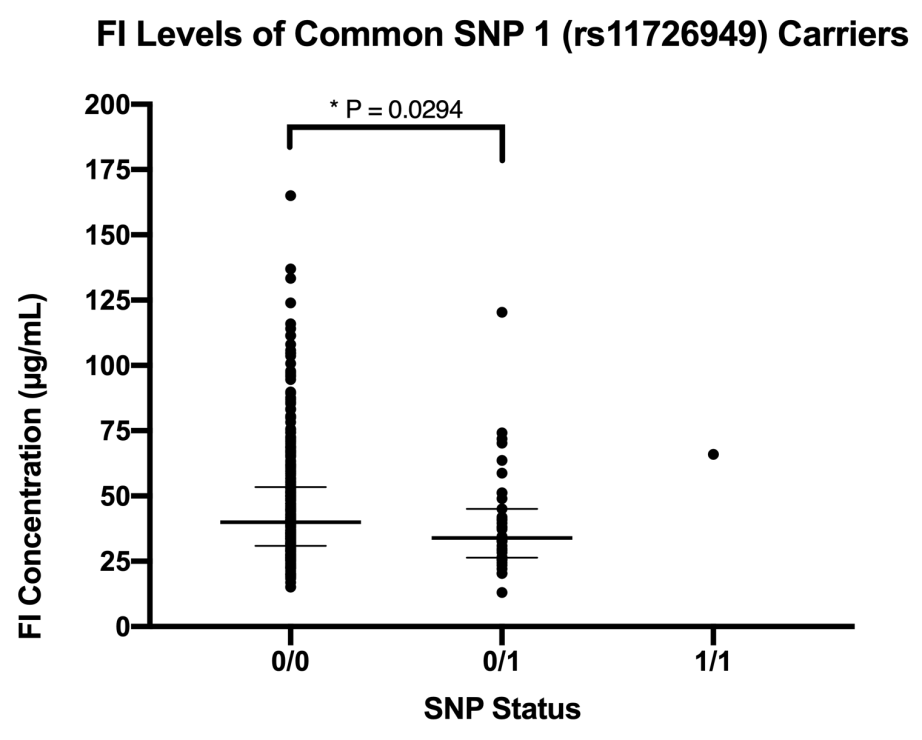
**

S2)

**
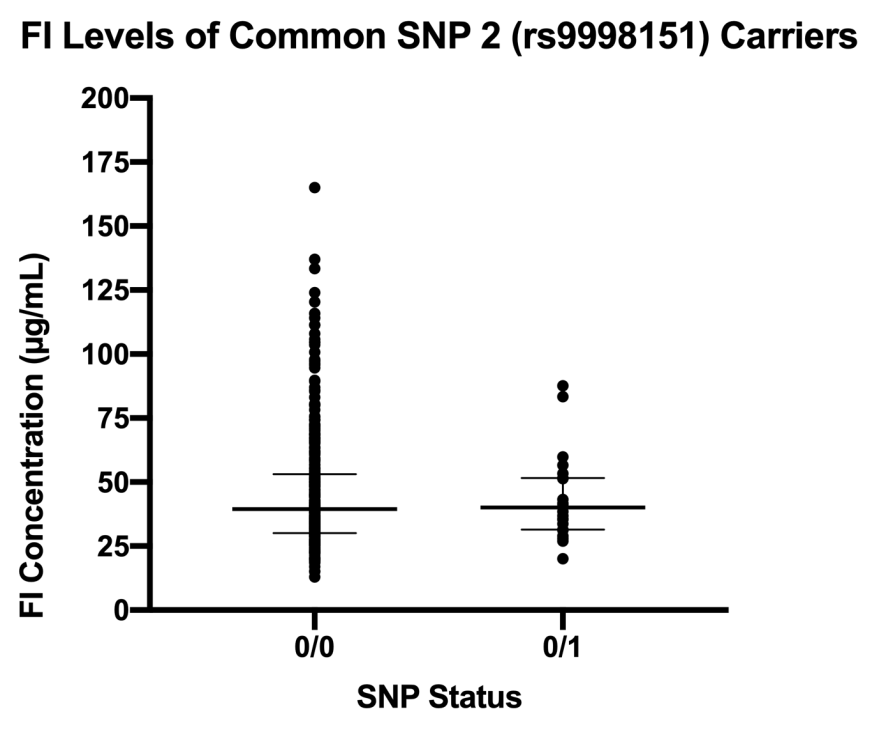
**

S3)

**Supplementary figures 2 and 3** - FI plasma levels of patients in the Southampton AMD cohort are plotted categorised by SNP status (SNP 1 (rs11726949) and SNP 2 (rs9998151)), previously associated with AMD in Fagerness et al., (2009).^1^ Individuals with a rare *CFI* variant were excluded from this analysis. Median with interquartile range is shown by bars. There was a small but significant difference in FI level when comparing carriers of SNP 1 in heterozygosity (0/1) compared to non-carriers (0/0) (P = 0.0294, for n of 39 and 341, respectively), however, there was no difference identified between carriers and non-carriers of SNP 2 (for n of 23 and 358, respectively). There were few patients homozygous for either SNP, limiting analysis. Further, there are small number of patients heterozygous for SNP 1 (n of 39) compared to non-carriers, which suggests this finding requires validation. *P > 0.05.

**
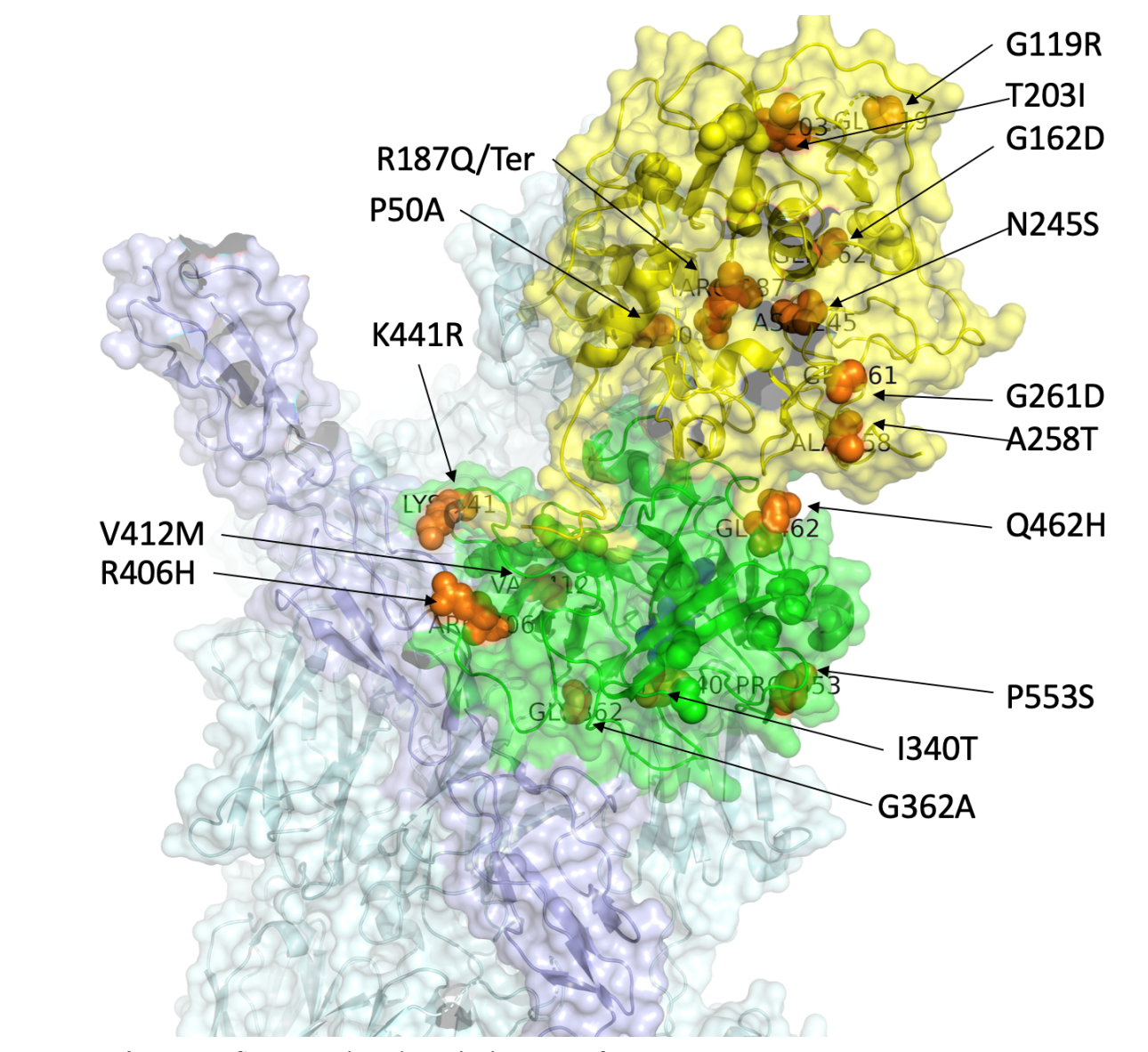
**

**Supplementary figure** 4 - Plotted are the locations of the identified rare genetic variants in FI in a 3D structure as it is bound to FH and C3b in the trimolecular complex. The heterodimer FI is visualized in green (light chain) and yellow (heavy chain), whereas FH CCP domains 1-4, which run through the centre, are in pale purple and C3b is in pale cyan. Each amino acid with identified variation in the cohort is displayed by orange spheres and labelled. Blue spheres in the centre of the light chain structure mark the catalytic triad. This graphic was produced using PyMOL (version 2.0.6, Schrödinger, LLC) and the PDB 5o23 molecular structure described in Xue et al., (2017).

**Supplementary Table** **1 -** *A complete analysis of each non-synonymous CFI variant identified in the Southampton AMD cohort.*

| ***HGVS Description*** | ***Polyphen-2 Score*** | ***Known Conditions*** | ***Functional Analysis / Systemic Effects*** | ***FI Serum/ Plasma Levels*** | ***Southampton AMD Cohort FI Levels*** | ***Position in 3D Pymol Structure*** |
| --- | --- | --- | --- | --- | --- | --- |
| *NM_000204.4:c.148C>G (P50A)* | *Probably Damaging* | *aHUS; AMD* | *High FB, impaired C3b and C4b degradation on surface, impaired C3b cleavage when surface bound.*^3^ | *Both normal and low (40µg/mL) serum levels, reduced secretion compared to WT.*^4^ *Secreted as well as WT, impaired activity on surface.*^3^ *Low FI levels in 1 patient with AAMD.*^5^ | *Normal level, 1 case, mixed AMD* | *Buried* |
| *NM_000204.4:c.355G>A (G119R)* | *Probably Damaging* | *aHUS; AMD* | *Lower C3b degradation related to low secretion.*^6^ | *Low serum levels,*^5^ *and secretion from HEK293 cells.*^7^ | *6 low, 1 normal level, 1 no plasma, 3 mixed AMD, 1 early AMD, 1 CNV, 1 GA, 1 control* | *Surface - no interaction* |
| *NM_000204.4: c.485G>A (G162D)* | *Probably Damaging* | *aHUS; AMD; FI Deficiency* | *N/D* | *Low serum levels.*^5,8^ *Causes clinical FI deficiency when in compound heterozygosity.*^9^ | *Low level, 1 case, mixed AMD* | *Buried* |
| *NM_000204.4:c.559C>T (R187*)* | *NA - Loss of Function* | *FI Deficiency; AMD* | *N/D* | *Low FI levels, causes clinical FI deficiency when in compound heterozygosity.*^9^ | *Low level, 1 case, early AMD* | *NA* |
| *NM_000204.4:c.560G>A (R187Q)* | *Possibly Damaging* | *MPGN_HUS; aHUS; AMD* | *N/D* | *N/D* | *Normal level, 1 control* | *Buried* |
| *NM_000204.4:c.608C>T (T203I)* | *Possibly Damaging* | *aHUS; MPGN; AMD* | *N/D* | *N/D* | *Normal level, 1 control* | *Buried* |
| *NM_000204.4:c.734A>G (N245S)* | *Probably Damaging* | *None* | *N/D* | *N/D* | *Low level, 1 case, CNV* | *Buried* |
| *NM_000204.4:c.772G>A (A258T)* | *Possibly Damaging* | *FI Deficiency; AMD* | *N/D* | *Causes clinical FI deficiency when in homozygosity.*^9^ | *Low level, 1 case, early AMD* | *Surface - no interaction* |
| *NM_000204.4:c.782G>A (G261D)* | *Benign* | *aHUS; C3GN; MPGN; AMD*^10^ | *Normal Function in the fluid phase and when surface bound.*^11,12^ | *Normal serum levels and secretion.*^5,12^ | *All normal levels, 6 cases, 8 controls, 3 mixed AMD, 2 early AMD, 1 GA* | *Surface - no interaction* |
| *NM_000204.4:c.1019T>C (I340T)* | *Probably Damaging* | *aHUS; AMD* | *Normal C3 serum levels*^13^ *Normal secretion, impaired fluid phase co-factor activity.*^12^ | *Normal serum levels.*^12^ | *Low level, 1 control* | *Buried - C3b binding site* |
| *NM_000204.4:c.1085G>C (G362A)* | *Benign* | *AMD* | *N/D* | *1 patient with normal levels, non-AAMD.*^5^ | *Low level, 2 cases, 2 CNV* | *Surface - close to C3b and FH* |
| *NM_000204.4:c.1217G>A (R406H)* | *Benign* | *aHUS; AMD* | *Normal secretion and functional fluid phase co-factor activity.*^12^ | *Normal serum levels.*^5^ | *Normal level, 1 CNV* | *Surface - FH binding site* |
| *NM_000204.4:c.1234G>A (V412M)* | *Probably Damaging* | *AMD; aHUS* | *N/D* | *Normal serum levels, 1 aHUS patient.*^14^ | *Low level, 1 case, early AMD* | *Surface - close to C3b and FH* |
| *NM_000204.4:c.1322A>G (K441R)* | *Benign* | *aHUS; MPGN; AMD;* | *N/D* | *Normal serum levels, 11% low.*^5^ | *2 low, 4 normal levels, 4 cases, 2 controls, 3 early AMD, 1 GA* | *Surface - FH binding site* |
| *NM_000204.4:c.1386A>T (D462H)* | *Benign* | *AMD; PE* | *N/D* | *1 control near bottom of normal range.*^15^ | *2 controls, near bottom of normal range (25 & 26µg/mL)* | *Surface – between FI heavy and light chain* |
| *NM_000204.4:c.1657C>T (P553S)* | *Benign* | *aHUS; MPGN; AMD* | *Impaired ability to degrade C3b* ^6^ | *Normal serum levels.*^5,6^ | *All normal levels, 2 cases, 3 controls, 1 early AMD, 1 mixed AMD* | *Surface - close to C3b and FH* |

*Every non-synonymous CFI variant was fully interrogated using previous literature and Pymol (v2) modelling. AAMD; advanced AMD, aHUS; atypical haemolytic uraemic syndrome, C3GN; C3 glomerulopathy, CNV; choroidal neovascularisation, FH; factor H, FI; factor I GA; geographic atrophy, MPGN; membranoproliferative glomerulopathy, N/A; not applicable, N/D; not done, PE; preeclampsia.*

Supplementary References:

1. Fagerness J a, Maller JB, Neale BM, Reynolds RC, Daly MJ, Seddon JM. Variation near complement factor I is associated with risk of advanced AMD. *Eur J Hum Genet*. 2009;17(1):100-104.

2. Xue X, Wu J, Ricklin D, et al. Regulator-dependent mechanisms of C3b processing by factor i allow differentiation of immune responses. *Nat Struct Mol Biol*. 2017;24(8):643-651.

3. Nilsson SC, Kalchishkova N, Trouw LA, Fremeaux-Bacchi V, Villoutreix BO, Blom AM. Mutations in complement factor I as found in atypical hemolytic uremic syndrome lead to either altered secretion or altered function of factor I. *Eur J Immunol*. 2010;40(1):172-185.

4. Bienaime F, Dragon-Durey M-A, Regnier CH, et al. Mutations in components of complement influence the outcome of Factor I-associated atypical hemolytic uremic syndrome. *Kidney Int*. 2010;77(4):339-349.

5. Kavanagh D, Yu Y, Schramm EC, et al. Rare genetic variants in the CFI gene are associated with advanced age-related macular degeneration and commonly result in reduced serum factor I levels. *Hum Mol Genet*. 2015;24(13):3861-3870.

6. Geerlings M, Kremlitzka M, Bakker B, Al E. The Functional Effect of Rare Variants in Complement Genes on C3b Degradation in Patients With Age-Related Macular Degeneration. *JAMA Ophthalmol*. 2017;135(1):39-46.

7. van de Ven JPH, Nilsson SC, Tan PL, et al. A functional variant in the CFI gene confers a high risk of age-related macular degeneration. *Nat Genet*. 2013;45(7):813-817.

8. Le Quintrec M, Lionet A, Kamar N, et al. Complement mutation-associated de novo thrombotic microangiopathy following kidney transplantation. *Am J Transplant*. 2008;8(8):1694-1701.

9. Alba-Domínguez M, López-Lera A, Garrido S, et al. Complement factor I deficiency: a not so rare immune defect. Characterization of new mutations and the first large gene deletion. *Orphanet J Rare Dis*. 2012;7(42):1-8.

10. Servais A, Frémeaux-Bacchi V, Lequintrec M, et al. Primary glomerulonephritis with isolated C3 deposits: a new entity which shares common genetic risk factors with haemolytic uraemic syndrome. *J Med Genet*. 2007;44(3):193-199.

11. Nilsson SC, Karpman D, Vaziri-Sani F, et al. A mutation in factor I that is associated with atypical hemolytic uremic syndrome does not affect the function of factor I in complement regulation. *Mol Immunol*. 2007;44(8):1835-1844.

12. Kavanagh D, Richards A, Noris M, et al. Characterization of mutations in complement factor I (CFI) associated with hemolytic uremic syndrome. *Mol Immunol*. 2008;45(1):95-105.

13. Bresin E, Rurali E, Caprioli J, et al. Combined Complement Gene Mutations in Atypical Hemolytic Uremic Syndrome Influence Clinical Phenotype. *J Am Soc Nephrol*. 2013;24:475-486.

14. Osborne AJ, Breno M, Borsa NG, et al. Statistical validation of rare complement variants provides insights on the molecular basis of atypical haemolytic uraemic syndrome and C3 glomerulopathy. *J Immunol*. 2018;200(7):2464-2478.

15. Seddon JM, Yu Y, Miller EC, et al. Rare variants in CFI, C3 and C9 are associated with high risk of advanced age-related macular degeneration. *Nat Genet*. 2013;45(11):1366-1370.
